# Fatal Methanol Poisoning Caused by Drinking Industrial Alcohol: Silesia Region, Poland, April–June 2022

**DOI:** 10.3390/toxics10120800

**Published:** 2022-12-19

**Authors:** Marcin Tomsia, Małgorzata Głaz, Joanna Nowicka, Julia Cieśla, Maciej Sosnowski, Elżbieta Chełmecka

**Affiliations:** 1 Department of Forensic Medicine and Forensic Toxicology, Medical University of Silesia, 18 Medyków Street, 40-752 Katowice, Poland; 2 Students Scientific Society, Medical University of Silesia, 18 Medyków Street, 40-752 Katowice, Poland; 3 Department of Statistics, Department of Instrumental Analysis, Faculty of Pharmaceutical Sciences, Medical University of Silesia, Ostrogórska 30, 41-200 Sosnowiec, Poland

**Keywords:** gas chromatography–flame ionization detector (GC-FID), formic acid, methanol, poisoning

## Abstract

Methanol poisonings caused by drinking industrial alcohol remain a severe problem worldwide. Education on types of alcohol and their harmfulness and legal regulations limiting the industrial alcohol trade seem to be the keys to reducing the number of poisonings. Methanol distribution in different tissues after absorption is not well understood. This research aimed to quantify the methanol and formic acid distribution in body fluids and tissue material in post-mortem samples collected from 19 fatal victims of massive intoxication with industrial alcohol in the Silesia Region (Poland) who died between April and June 2022. The samples were analyzed using a gas chromatography–flame ionization detector (GC-FID), and correlation coefficients for methanol and formic acid were determined. The results show a wide distribution of methanol and formic acid in human post-mortem biological fluids (blood, urine, vitreous humor, bile, and cerebrospinal fluid) and tissues (muscle, kidney, liver, spleen, lung, and brain). The strongest correlation for methanol concentration in blood and body fluids/tissues was obtained in the cerebrospinal fluid (r = 0.997) and for formic acid in muscle tissue (r = 0.931). The obtained results may be a valuable tool in toxicological analysis and improve medical standards of early diagnosis and targeted treatment.

## 1. Introduction

Methanol (CH_3_OH), also known as methyl alcohol or wood alcohol, is commonly used as a solvent in different branches of industry [1,2]. Methanol poisoning by ingestion is a global problem, and it is closely related to high morbidity and mortality [3]. Alcohol dehydrogenase oxidizes methanol to formaldehyde, and aldehyde dehydrogenase subsequently oxidizes formaldehyde to formic acid, which accounts for the associated anion gap metabolic acidosis and end-organ damage [4]. Therefore, methanol ingestion can be fatal. Pure methanol lethal dose is estimated to range from 300 to 1000 mg/kg [5]. Although the recommendations of the American Academy of Clinical Toxicology practice guidelines on the treatment of methanol poisoning are already 20 years old [6], they are still valid because mass poisonings with beverages containing methanol occur all over the world [7,8,9]. Methanol poisoning carries numerous health consequences, which are often irreversible. One of the most characteristic symptoms of methanol poisoning is eyesight damage in the form of induced optic neuropathy. There are currently no effective treatment methods for this disease due to the multifactorial aspects of eye damage [10]. However, Luo et al. [11] reported the successful recovery of visual abilities in three fireworks factory workers who inhaled the methanol. The recovery was possible due to instant diagnosis and treatment that included hemodialysis [11]. The range of possible complications after exposure to methanol is wide. The literature describes, among others, rare cases of diabetic ketoacidosis [12], parkinsonism, and cerebral vasculopathy [13]. Currently used strategies for treating methanol poisoning include: administering the substances competing with methanol for alcohol dehydrogenase (ethyl alcohol, fomepizole) metabolism, increasing formaldehyde (folinic acid) metabolism [14], and hemodialysis [14,15]. From the above-mentioned substances, fomepizole is the most important antidote for methanol poisoning due to its higher than methanol affinity for alcohol dehydrogenase [16]. Studies show that delayed initiation of appropriate treatment and low value of the Glasgow Coma Scale (GCS) are the main causes of increased mortality in methanol-poisoned patients [17,18].

The ongoing reports on newly discovered consequences of methanol poisoning result from the difficulties in identifying methanol poisoning symptoms, their non-specificity, and the lack of standard methanol blood testing. Furthermore, the distribution of methanol in different tissues and body fluids after absorption is not well understood. Our research aimed to determine the distribution of methanol and formic acid in body fluids and tissues sampled from 19 victims of fatal massive intoxication with industrial alcohol who died between April and June 2022 in the Silesia Region (Poland).

## 2. Materials and Methods

The study material was collected from 19 individuals who died of methanol poisoning between April and June 2022. According to media reports, the most likely source of methanol was denatured alcohol ingestion. Tissue and body fluids sampling was approved by the Bioethical Commission of the Medical University of Silesia in Katowice (decision no. PCN/CBN/0052/KB/77/22, date of approval: 5 May 2022). Femoral blood, urine, vitreous humor, cerebrospinal fluid, bile, muscle, liver, kidney, and spleen samples were collected during medico-legal autopsies commissioned by the Prosecutor’s Office. All analyses were carried out in a certified forensic laboratory immediately after the autopsy.

The collected samples, 1 mL of fluid or 1 g of chopped tissue, were placed in 20 mL glass vials and analyzed using a Focus GC gas chromatograph equipped with a Triplus autosampler (oven temperature of 60 °C, equilibrating time 5 min, injection volume 1.4 mL), FID detector (Thermo Fisher Scientific Inc., Milan, Italy) and Rtx^®^–BAC2 column (30 m × 0.53 mm ID × 2.0 μm) (Restek Corp., Bellefonte, PA, USA). The oven temperature program was 45 °C (5 min), 45–80 °C (10 °C/min), 80 °C (1 min). Injector and detector temperatures were 200 °C and 250 °C, respectively. The carrier gas was helium (5.0 mL/min). Tert-butyl alcohol (1 mg/L) was used as an internal standard (0.2 mL). An eight-point calibration curve for methanol in mg/mL or mg/g (0; 0.1; 0.2; 0.5; 0.8; 1; 2; 3) was linear in the whole range. The limit of detection (LOD) was determined as 0.05 mg/mL or mg/g, and the limit of quantification (LOQ) as 0.1 mg/mL or mg/g for the entire tested material. Linearity was maintained up to 5000 mg/L (R^2^ = 0.996).

Formic acid concentration in blood, urine, and tissues was determined using gas chromatography and the method described by Kuo et al. [19] and Abolin et al. [20]. In this method, formic acid was determined in the form of a volatile methyl formate ester. Using FID detector (Thermo Fisher Scientific Inc., Milan, Italy) allowed for a sensitivity of 0.01 mg/mL and minimized the impact of the biological background.

The distribution of variables was evaluated by the Shapiro–Wilk test and quantile-quantile plot. The interval data were expressed as a mean value ± standard deviation. The regression analysis was used to determine the relationship between quantitative features. In the linear regression model, the existence of outlier data was verified with Cook’s distances based on the residuals. Statistical significance was set at a *p* < 0.05, and all tests were two-tailed. Statistical analysis was performed using Statistica version 13.3 (TIBCO Software Inc., Palo Alto, CA, USA, 2017).

## 3. Results

The study group consisted of 3 women (16%) and 16 men (84%). Only 5 individuals (26%) were hospitalized. The basic characteristics of quantitative variables are presented in the Table 1.

In the case of hospitalized individuals, the results differed from the rest of the study group. In three subjects, methanol was not found in all tissues, which most likely resulted from the methanol flushing during hospital treatment (the time from the last consumption of contaminated alcohol to death was 5, 12, and 12 days, respectively). However, the concentration of formic acid in the tissues was detected in these individuals. Formic acid in two locations: the cerebrospinal fluid and the brain, was detected in only one of them. The remaining two hospitalized individuals had traces of methanol concentrations in the brain and in the cerebrospinal fluid, while formic acid was detected in most tissues.

Disregarding the five individuals with zero methanol concentration in the blood, in the rest of the cases, the highest methanol concentration was observed in the urine (n = 10), while the lowest values were observed in the liver (n = 6).

We found statistically significant correlations between methanol concentration in the blood and in other tissues and between formic acid concentration in the blood and in other tissues except for the kidneys (Table 2).

The blood-to-urine ratio for methanol concentration was calculated for all non-hospitalized individuals (Table 3).

These results show that most people did not die immediately after consuming industrial alcohol. In one individual, the methanol blood-to-urine ratio was surprisingly high (1.90), which indicated fresh alcohol consumption. Unfortunately, in this case, the information about the time that elapsed from consumption to death (t1) was unavailable, which is why this case was excluded from Table 1.

## 4. Discussion

The presented research contributes to the current knowledge on methanol distribution in the human body and may have clinically important consequences. Most of the published studies about mass methanol poisonings usually analyze the hospitalized patients poisoned with methanol, while we present the results of the post-mortem studies.

The cases describing the deceased with no methanol detected in the tissues but only in the brain and cerebrospinal fluid are clinically important. They confirm the assumption proposed by Andersen et al. [21], who recommended post-mortem analysis of the brain in the deceased with a prolonged survival time. We suggest that in the case of late methanol poisoning discovery, in patients who have undergone hemodialysis/gastrointestinal lavage or in cases of dispute, cerebrospinal fluid may also be analyzed.

We also showed methanol distribution in individual tissues. This gives another insight into methanol metabolism since metabolized methanol is insufficiently cleared by the kidneys or lungs [22]. Our results indicated samples where methanol accumulated in a wider cohort and confirmed its presence in the body even days after consumption. It may be important in the context of possible late organ complications in people poisoned with sublethal methanol doses.

The methanol partition coefficient in biological fluids and tissues were analyzed, and the maximum values were found in the liver (1.27 ± 0.24), while the lowest were in urine (0.76 ± 0.09). In the case of ethanol, Van Hecke et al. [23] showed opposite results, with the maximum values noted in urine (1.33) and the minimum values noted in the liver (0.52) probably resulting from the ethanol elimination from the body. The comparison of the methanol and ethanol [23] partition coefficients showed higher partition coefficient values for methanol in the tissues but lower in the body fluids. Since the body fluids contain more water than the tissues and methanol is more soluble in water than ethanol, the presented results compare to the results obtained for ethanol as expected.

Mass fatal intoxications with methanol remain a serious problem in least developed, developing, and middle-developed countries. Our study is the first to describe the incident of mass methanol poisoning in Poland. Among other publications, there is only one description of a similar series of deaths, in the Czech Republic [9].

Our study also revealed formic acid presence in the tissues, which is important in the context of poisoning complication prevention in survivors. Zakharov et al. [24] reported possible carcinogenic effects of methyl alcohol exposure six years earlier that related to the oxidation of this alcohol in the tissues in the course of fatal mass poisoning in the Czech Republic [24]. Therefore, based on our findings on distribution, it is imperative to continue this type of prospective study to understand the full profile of possible late complications in survivors of methanol poisoning.

Recent outbreaks of mass methyl alcohol poisoning were observed in Tehran, Saudi Arabia, and India [25,26,27]. The descriptions of these poisonings differ primarily in the ratio of poisoned women to poisoned men. The outbreak we described included 3 women out of 19 dead, while in a similar case in India, only men were poisoned [27]. In the case of the outbreak in Saudi Arabia, there were almost as many women (4) as men (5) [26]. However, all the reported cases include too few poisoned and dead for an accurate assessment of the risk of exposure and the clinical course between the sexes.

Recently, the number of methanol poisonings has increased worldwide. One of the reasons that researchers describe is the COVID-19 pandemic. Mousavi-Roknabadi et al. [28], in a meta-analysis published this year, listed several possible causes, including lack of education about types of alcohol and the risks associated with their consumption, methods of preventing COVID-19 infection, and the prevalence of disinfectants based on various types of alcohol. Also, legal regulations in some countries make the trade in non-consumer alcoholic beverages more and more easier [28].

In addition to the social dimension, the presented case should be an incentive for broader education and awareness in medical personnel. According to Issa et al., it is necessary to educate doctors and nurses about methanol poisoning symptoms and treatment, increase the role of toxicologists, and update the guidelines more frequently [29].

The main limitation of the present study is the lack of access to patients’ medical records and the lack of information on the exact amount of methanol consumed. Future research should focus on possible predictors of the risk of death from methanol poisoning. Abdelwahab et al., proposed to use the neutrophil-to-lymphocyte ratio and the platelet-to-lymphocyte ratio as predictors for acute methanol poisoning [30]. Future research must focus on the early symptoms of methanol poisoning and carefully examine the changes in the body that occur immediately after ingestion. Delays in treatment are the main cause of therapeutic failure, especially considering that the first 24 h after consuming methanol may be asymptomatic [31]. Since the research on determining volatile organic compounds in commercial alcoholic beverages is ongoing [32,33], establishing procedures for the qualitative and quantitative control of volatile substances in industrial alcohols should be considered.

## 5. Conclusions

Our study showed a wide distribution of methyl alcohol in the human body. The results indicate the presence of methanol and formic acid in body fluids and tissues many days after death, which may be a valuable tool in a toxicological analysis and in the search for the cause of death in cases of corpses with prolonged post-mortem interval (PMI).

## Figures and Tables

**Table 1:** Descriptive statistics of quantitative variables analyzed in the victims of fatal methanol poisoning (N = 19).

Variable	N	Mean	SD	Median	Q_1_	Q_3_	x_min_	x_max_
Age	17	50.8	12.1	49.0	43.0	61.0	33.0	74.0
t1 [days]	15	5.8	4.9	3.0	1.0	12.0	1.0	13.0
t2 [days]	17	7.2	3.4	7.0	6.0	8.0	1.0	16.0
**Methanol [mg/mL or mg/g]**								
Blood	19	2.45	1.81	2.61	0.00	4.28	0.00	5.14
Urine	17	3.36	2.52	3.30	2.20	5.44	0.00	8.20
Vitreous humor	12	3.26	2.00	3.21	2.09	5.31	0.00	5.78
Cerebrospinal fluid	14	3.30	2.49	3.44	0.01	5.43	0.00	6.74
Bile	16	2.80	2.34	2.81	0.00	5.26	0.00	6.13
Muscle *	16	2.22	1.70	2.50	0.00	3.63	0.00	4.49
Liver *	17	2.02	1.52	2.41	0.00	2.57	0.00	4.40
Kidney *	13	3.42	1.49	3.68	2.61	4.63	0.00	5.24
Spleen *	17	2.39	1.78	2.36	0.00	3.62	0.00	4.73
**Formic acid [mg/mL or mg/g]**								
Blood	19	0.77	0.51	0.95	0.03	1.16	0.00	1.49
Urine	16	2.94	2.65	3.09	0.06	4.73	0.00	8.36
Vitreous humor	12	0.65	0.47	0.66	0.29	0.93	0.00	1.52
Cerebrospinal fluid	13	0.43	0.38	0.37	0.06	0.68	0.01	1.15
Bile	15	0.76	0.55	0.85	0.08	1.24	0.00	1.63
Muscle *	16	0.44	0.32	0.46	0.08	0.74	0.00	0.88
Liver *	17	0.49	0.52	0.38	0.01	0.87	0.00	1.79
Kidney *	13	0.67	0.44	0.66	0.38	1.05	0.02	1.24
Spleen *	17	0.50	0.46	0.40	0.01	0.83	0.00	1.56

Legend: *—substance tissue concentration expressed in mg/g, t1—time from the last consumption of an industrial alcohol to death, t2—time from death to autopsy, SD—standard deviation, Q_1_—lower quartile, Q_3_—upper quartile

**Table 2:** Analysis of univariate linear regression for methanol concentration in blood and other body fluids and tissues of fatal methanol poisoning victims.

Concentration in the Blood	Concentration in Other Fluids or Tissues	β	SE (β)	r	*p*
Methanol	Urine	1.304	0.134	0.929	<0.001
	Vitreous humor	1.173	0.044	0.993	<0.001
	Cerebrospinal fluid	1.288	0.030	0.997	<0.001
	Bile	1.112	0.119	0.929	<0,001
	Muscle	0.886	0.046	0.982	<0.001
	Liver	0.771	0.059	0.959	<0.001
	Kidney	1.030	0.073	0.974	<0.001
	Spleen	0.916	0.055	0.974	<0.001
	Lung	0.928	0.058	0.974	<0.001
	Brain	0.912	0.042	0.986	<0.001
Formic acid	Urine	3.990	0.852	0.781	<0.001
	Vitreous humor	0.735	0.215	0.734	<0.01
	Cerebrospinal fluid	0.569	0.139	0.776	<0.01
	Bile	0.891	0.151	0.853	<0.001
	Muscle	0.542	0.057	0.931	<0.001
	Liver	0.708	0.166	0.740	<0.001
	Kidney	0.597	0.306	0.507	0.077
	Spleen	0.706	0.124	0.827	<0.001
	Lung	0.803	0.135	0.846	<0.001
	Brain	0.449	0.099	0.795	<0.001

Legend: β—regression coefficient, SE (β)—standard error for the regression coefficient, r—Pearson’s linear correlation coefficient.

**Table 3:** The blood-to-tissue or body fluid partition coefficient for methanol detected in fatal methanol poisoning victims (n = 13). The results are presented as mean ± SD.

Tissue/Body Fluid	Blood-to-Tissue or Body Fluid Partition Coefficient
Urine	0.76 ± 0.09
Vitreous humor	0.86 ± 0.07
Cerebrospinal fluid	0.79 ± 0.04
Bile	0.86 ± 0.11
Muscle	1.08 ± 0.14
Liver	1.27 ± 0.24
Kidney	0.98 ± 0.11
Spleen	1.07 ± 0.15
Lung	1.07 ± 0.14
Brain	1.07 ± 0.11

## Data Availability

Not applicable.
